# Pasteurized Milk Harboring Diarrheagenic *E. coli* Virulence Genes: Biosurveillance and New Insights for Enhancing Food Safety Standards

**DOI:** 10.1155/vmi/3144493

**Published:** 2025-10-13

**Authors:** Samanta Stinghen de Abreu, Stael Málaga Carrilho, Aline Martins Mancebo, Marina Rocha Dorella, Edson Antônio Rios, Ronaldo Tamanini, Aline Romano Cunha, Ulisses de Pádua Pereira, Rafael Fagnani

**Affiliations:** ^1^Department of Veterinary Preventive Medicine, Universidade Estadual de Londrina-UEL, Londrina, Paraná, Brazil; ^2^Programa de Pós-graduação em Saúde e Produção Animal, Universidade Anhanguera Unopar, Arapongas, Paraná, Brazil

**Keywords:** coliforms, contamination, dairy, diarrhea, postpasteurization

## Abstract

Members of the family *Enterobacteriaceae* are often linked to foodborne outbreaks, including acute diarrhea, with diarrheagenic *Escherichia coli* (DEC) as the most common cause of this disease in low- and middle-income countries. A cross-sectional study was carried out to evaluate the sanitary conditions of ready-to-consume milk, involving 750 pasteurized milk samples. These samples were analyzed for the *Enterobacteriaceae* count, the presence of *Salmonella* spp., and the enumeration of *E. coli*, as well as checking for the presence of DEC-specific virulence genes. Two molecular assays were used to detect DEC-specific virulence genes (*eae, bfpA, aggR, ipaH, est, elt, stx1,* and *stx2*). Overall, a significant noncompliance rate (5%) was identified in the *Enterobacteriaceae* counts, indicating postpasteurization contamination. Two genes (*ipaH* and *elt*) were detected in 14.28% of *E. coli*-positive samples, highlighting the need for improved methods to minimize postpasteurization contamination in dairy plants. This improvement could contribute to better food safety standards internationally. Additionally, further studies are necessary to understand the actual risk posed by these strains circulating in milk for immunocompromised individuals or those with immature immune systems. This underscores a global concern for vulnerable populations worldwide.

## 1. Introduction

Monitoring *Salmonella* spp., *Enterobacteriaceae*, and *Escherichia coli* is an essential tool for evaluating enteric contamination along the dairy chain, especially in ready-to-eat products such as pasteurized milk [1].

These microorganisms are frequently involved in foodborne outbreaks, mainly related to acute diarrhea, which is one of the major public health issues worldwide, characterized by high morbidity and mortality rates, particularly in young children in developing countries. Data released by the World Health Organization (WHO) indicate that diarrhea is the second leading cause of childhood mortality [2].

According to the WHO, the most common agents causing acute diarrhea in low- and middle-income countries are rotavirus and diarrheagenic *E. coli* (DEC) [3]. DECs are significant foodborne pathogens with considerable importance for public health, grouped into six pathogenic types: Shiga toxin-producing *E. coli* (STEC), enteropathogenic *E. coli* (EPEC), enterotoxigenic *E. coli* (ETEC), enteroinvasive *E. coli* (EIEC), enteroaggregative *E. coli* (EAggEC), and diffusely adherent *E. coli* (DAEC) [4].

Fallah et al. (2021) [5] found a high prevalence (26%) of DEC strains, primarily from hamburger and cheese samples, indicating food contamination with intestinal pathogens during processing or postprocessing. Additionally, a study conducted in northwest Paraná demonstrated a high diversity of *E. coli* populations found in pasteurized milk, likely due to the wide dispersion of these bacteria in nature, with different sources of contamination that can be incorporated into the milk [6]. Another study characterized 56 EPEC strains isolated from three different pasteurized milk brands in Rio de Janeiro, concluding that the environmental strains studied exhibited atypical characteristics, distinct from those observed in clinical strains of human origin, yet still capable of causing lesions and being potentially pathogenic [7]. These results highlight the importance of ongoing surveillance and research on this microorganism in food, with milk often cited as a potential vehicle for the transmission of DEC [8].

Considering that the circulation of bacteria from the *Enterobacteriaceae* family in ready-to-eat products is not an unusual phenomenon [9, 10], this study aimed to assess the sanitary conditions of pasteurized milk and detect specific virulence genes of DEC strains using molecular assays through polymerase chain reaction (PCR).

## 2. Materials and Methods

### 2.1. Sampling

A cross-sectional study was performed on data collected from 750 pasteurized milk samples, all originating from different production batches, from January 2019 to November 2022. The sampling plan involved periodic quality control analyses of three dairies in Londrina and Arapongas, Paraná, Brazil, as well as market surveillance conducted by the Brazilian Health Regulatory Agency (ANVISA), covering pasteurized milk from 21 dairy plants across 13 municipalities in the north and northwest of Paraná.

According to the national regulations in effect at the time, dairy plants were required to submit at least three samples per month for microbiological monitoring, which defined the frequency and flow of sample submissions.

The sample size in this study reflects the milk samples routinely submitted between January 2019 and November 2022 to a university-based laboratory that serves as a regional reference center for dairy quality control. The sampling followed a convenience approach, based on the laboratory's routine case load during the study period.

No a priori sample size calculation was performed, as the study design was retrospective and based on the existing data from regulatory monitoring. Although the nonprobabilistic nature of this sampling limits the extrapolation of findings to broader populations, the substantial number of samples collected over nearly 3 years allows for a representative overview of milk quality conditions in the northern region of Paraná.

### 2.2. Analysis of Pasteurized Milk Samples

The enumeration of *Enterobacteriaceae* followed ISO 21528:2017 [11], and the survey for *Salmonella* spp. followed ISO 6785:2001 recommendations [12].

The quantitative determination of *E. coli* using the most probable number (MPN) followed the guidelines included in the ISO 7251:2005 [13]. All samples were tested for the alkaline phosphatase presence following the official methodology outlined by the Association of Official Agricultural Chemists [14].

The results from compositional and microbiological analyses were based on the percentage of samples that complied with the parameters set by the Brazilian statutory regulations for pasteurized milk.

In the confirmatory step of the analysis, samples cultivated in EC broth that fermented lactose and produced gas indicated the presence of viable *E. coli* cells and were then isolated. From EC broth positive tubes, two loopfuls of broth were streaked onto MacConkey agar (HiMedia, India) plates using the quadrant streak method and incubated at 37°C for 24 h. Colonies with typical *E. coli* morphology—round, pink/brick red (lactose-fermenting) or occasionally white, nonmucoid colonies—were selected (3–5 colonies per plate) and subcultured onto nutrient agar to ensure purity. These isolates were then subjected to standard biochemical tests, as described below.

The oxidase test was performed using oxidase reagent strips (Merck), where a portion of each colony was transferred onto the strip with a sterile wooden stick. The absence of color change within 30 s was interpreted as a negative result, consistent with *E. coli*. Indole production was assessed using SIM medium, which was inoculated with a single colony and incubated at 37°C for 24–48 h. After incubation, Kovac's reagent was added, and the appearance of a red layer at the surface indicated a positive indole reaction. The methyl red (MR) and Voges–Proskauer (VP) tests were performed in MR-VP broth incubated at 37°C for 48 h. For the MR test, the addition of five drops of the MR indicator resulting in a red coloration was considered a positive result, indicating stable acid production from glucose fermentation. For the VP test, 15 drops of Barritt's reagent A (α-naphthol) and 5 drops of reagent B (KOH) were added to the same broth. A red color developing within 30 min indicated a positive result; however, *E. coli* typically yields a negative VP result. Finally, citrate utilization was evaluated using Simmons citrate agar slants. The slants were streaked and incubated at 37°C for 24–48 h. A color change from green to blue indicated citrate utilization, while no color change (retaining the green color) was considered a negative result, as expected for *E. coli*. Confirmed *E. coli* strains were selected for DNA extraction and subsequent molecular analysis.

### 2.3. Preparation of DNA Samples and PCR Amplification

DNA extraction from samples with cultivated *E. coli* was performed using the boiling method, according to Ribeiro Junior [15], with modifications. Phosphate-buffered saline (PBS) and Tris-HCl EDTA (TE) were used as reagents. To obtain the bacterial mass, 1000 μL of broths containing *E. coli* were pipetted into microtubes, which were then centrifuged (14,500 rpm, 5 min). The supernatant was discarded, and the precipitate was treated with the aforementioned reagents, followed by boiling (100°C, 15 min) and cooling in an ice bath (15 min). Finally, a second centrifugation was performed (14,500 rpm, 10 min), and 50 μL of the supernatant was recovered for use in molecular analyses. This protocol ensures that the bacterial mass from *E. coli* cultivated in the broth was specifically targeted for DNA extraction.

The aliquots containing extracted genetic material were subjected to molecular assays to detect eight specific virulence genes of DEC. The selected targets for investigation and possible classification in groups were the *eae* and *bfpA* genes for EPEC, the *aggR* gene for EAggEC, *ipaH* gene for EIEC, *est* and *elt* genes for ETEC, and *stx1* and *stx2* genes for STEC.

The assays adopted were performed according to Fujioka et al. [16]. The first assay included the detection of *eae*, *bfpA*, and *aggR* genes and involved preparing a 20-μL reaction mix with 10x Tris-HCl buffer (200 mM Tris-HCl, 50 mM KCl, pH 8.4), 2 mM MgCl_2_, 2.5 μM of each phosphorylated deoxynucleotide, 20 μM of each primer, 1.25 U of Taq DNA polymerase (Invitrogen), and a final DNA volume of 5 μL. The second assay was used for the detection of *ipaH*, *est*, *elt*, *stx1*, and *stx2* genes and was performed with a 24-μL reaction mix containing 10x Tris-HCl buffer (200 mM Tris-HCl, 50 mM KCl, pH 8.4), 1.5 mM MgCl_2_, 2.5 μM of each phosphorylated deoxynucleotide, 20 μM of each primer, 1.5 U of Taq DNA polymerase (Invitrogen), and a final DNA volume of 1 μL.

**Figure 1:**
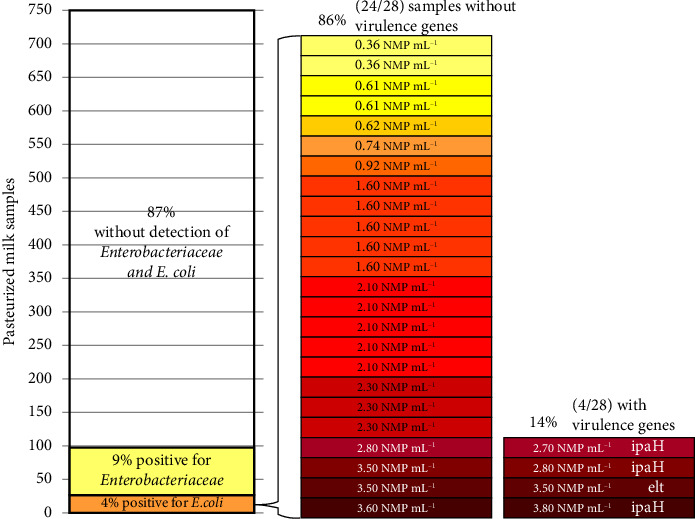
Relative and absolute frequencies of detection of *Enterobacteriaceae* and *E. coli* in 750 pasteurized milk samples, including *E. coli* microbiological load (MPN mL^−1^) and the identification of virulence genes, assessed from 2019 to 2022.

To ensure the validity and reproducibility of the PCR results, each reaction set included both a positive and a negative control. Positive controls consisted of DNA extracted from *E. coli* reference strains known to harbor the target virulence genes (as shown in Figure 1), while negative controls used ultrapure water instead of DNA template to monitor contamination.

The *E. coli* reference strains used in this study were as follows: *E. coli* EIEC O152 (ipaH-positive; labcode E23), *E. coli* ETEC H302 (elt and est-positive; labcode E12), *E. coli* STEC H301 (stx1-positive; labcode E17), and *E. coli* STEC 43889 (stx2-positive; labcode E6). All strains were obtained from the existing culture collection of the Laboratory of Inspection of Products of Animal Origin (LIPOA), State University of Londrina (UEL), Brazil. These controls were included in every PCR run, following standard laboratory quality assurance protocols.

The reactions followed amplification in the thermal cycler ThermoAssist Aeris-BD048 (Esco Micro Pte), and the reaction products were separated by electrophoresis (100 V, 60 min, LPS Power Supply 300 V, Loccus do Brasil) in 1.5% agarose gel containing ethidium bromide. The results were read in darkroom of an ultraviolet transluminator (UVCI-1200, Major Science).

Details about the primers employed in the study are in Table 1. All the oligonucleotides were synthesized by Invitrogen (Thermo Fisher Scientific, Brazil).

Descriptive statistical analysis was conducted to evaluate the absolute and relative frequencies of virulence genes across the samples. Binary logistic regression was also used to assess whether the *E. coli* load influences the likelihood of virulence genes being present. All statistical analyses were performed using Statistica software 13.0 (Statsoft, USA), with *p* < 0.05 considered statistically significant.

## 3. Results and Discussion

**Figure 2:**
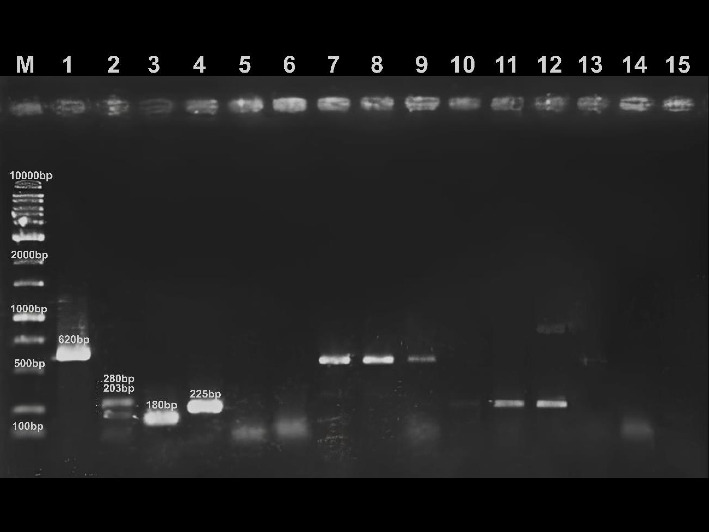
Agarose gel electrophoresis with specific bands and size (in base pairs) obtained from the amplification of the target genes. Lane M: molecular weight marker (1000 bp ladder, Kasvi, Brazil). Lane 1: *E. coli* positive for the *ipaH* gene (EIEC). Lane 2: *E. coli* positive for the *elt* and *est* genes (ETEC). Lane 3: *E. coli* positive for the *stx1* gene (STEC). Lane 4: *E. coli* positive for the *stx2* gene (STEC). Lanes 5–6: negative samples. Lanes 7–9: positive samples for the *ipaH* gene. Lane 10: negative sample. Lanes 11–12: positive sample for the *elt* gene. Lanes 13 and 14: negative samples. Lane 15: negative control.

Of 750 pasteurized milk samples analyzed during the period, 68 (9%) were positive for *Enterobacteriaceae*, 28 (4%) for *E. coli*, and no sample showed the presence of *Salmonella.* The relative and absolute frequencies of detection of *Enterobacteriaceae* and *E. coli*, along with the *E. coli* microbiological load (MPN mL^−1^), are detailed in Figure 2. Approximately 5% of the samples exceeded the Brazilian statutory limit of 10 CFU·mL^−1^ for *Enterobacteriaceae* (Table 2). The mean values and standard deviations for all microorganism counts are presented in Table 2. All noncompliant milk samples were negative for alkaline phosphatase, indicating contamination after pasteurization.

These findings align with global research, reinforcing the fact that postpasteurization contamination remains a common concern for dairy processors worldwide [21]. To better illustrate this issue, around 50% of pasteurized fluid milk spoilage in the United States of America is attributed to postpasteurization contamination [22]. Flaws in cleaning and sanitization protocols, cross-contamination, and absence of preventive maintenance are the main reasons for this kind of issue. Thus, efforts are needed to reduce postprocessing contamination and to identify its causative factors, mainly through approaches such as root-cause analysis and/or source tracking of contamination. Recently, predictive models and digital tools have been developed to support improved decision-making regarding postprocessing contaminations interventions, concluding in a good and cost-effective approach for enhancing fluid milk quality [23].

A number of studies indicate contamination of pasteurized milk by *Enterobacteriaceae* family members, including total coliforms and *E. coli* [24, 25], with the formation of resistant biofilms in the equipment pipes and the use of nonpotable water being the main factors responsible for the contaminations. Biofilms' formation is a constant concern in food factories, since they cause serious hygienic problems and generate deterioration of the products, also configuring economic losses [26].

The frequency of contamination with DEC genes in all 28 samples contaminated with *E. coli* was 14.28% (4/28) (Figure 2). The genes detected were *ipaH* (10.71%) and *elt* (3.57%) (Figure 1). Binary logistic regression revealed that the *E. coli* load is a significant predictor of virulence genes, with the likelihood of these genes occurring in pasteurized milk increasing 7.21 times for each additional unit of *E. coli* (MPN mL^−1^) (Table 3).

Due to the low frequency of samples with DEC genes, it was not possible to identify risk factors associated with the presence of these genes through statistical analyses such as binomial logistic regression and/or cluster analysis. Of the 28 samples contaminated with *E. coli*, the 4 that tested positive for DEC genes came from different dairies, with no single establishment being responsible. All 24 dairies involved in this study utilized high temperature and short-time (HTST) pasteurization. No differences were identified in pasteurization procedures between the dairies with positive samples and those without detection of DEC genes; however, it is reasonable to assume that a deeper investigation into the processing practices and quality control of each dairy could provide additional insights. With a larger collection of dairies having positive samples for DEC genes, it would be possible to identify which variables are related to this type of contamination, representing a relevant aspect for future studies.

Although the results improved upon previous publications, some limitations need to be considered. Initially, DNA-based tests can identify both living and nonliving pathogens, which can be beneficial given the cultivable characteristics of *E. coli*, such as being time-consuming and labor-intensive. However, this can also lead to some disadvantages. Detecting genetic material from nonviable cells can result in positive test results. To minimize this type of bias, the protocol includes a confirmatory step that focuses on assessing cell viability prior to DNA extraction. By employing selective media that enriches viable cells, the aim was to enhance the accuracy of findings and reduce the likelihood of false positives associated with nonviable genetic material.

Thus, the presented results do not detract from the fact that there is circulation of DEC strains in the dairy chain, inspires ongoing investigation to trace the origin of this contamination, possibly stemming from bacteria in raw milk, consequently causing cross-contamination in the dairy plants. Additional studies, such as strain isolation and characterization, will be essential to confirm these hypotheses and deepen the understanding of the associated risks.

The expression of virulence factor genes can be affected by environmental factors, such as pH, temperature, osmolarity, short-chain fatty acids, glucose, oxygen concentration, and probiotics. All these variables can be found in pasteurized milk, and how they interact with the expression of virulence genes could be evaluated by further studies. In addition to the reduced shelf life caused by the presence of coliforms in milk, it is necessary to discuss the sanitary risk associated with the presence of *E. coli* strains, which can become part of contaminating microbiota in dairy plants, acquiring and transferring virulence genes, and developing pathogenic potential.

The possibility of gene transfer involving the pathogenic strains of *E. coli* is a phenomenon that can easily occur in biofilms. *E. coli* is one of the more versatile bacteria adopting genetic information for secreting virulence genes by horizontal gene transfer, conferring pathogenic properties on innocuous strains [27]. Virulence genes are often located in regions called pathogenicity islands (PAIs), characterized by large size (> 10 kb), usually adjacent to tRNA genes, flanked by repeated sequences and may be unstable regions, and are considered to have evolved through horizontal gene transfer. An example of this genetic phenomenon occurring in practice is an outbreak of hemolytic uremic syndrome and bloody diarrhea started in Germany caused by a hybrid pathogenic EAEC-STEC strain (O104:H4), with most of the isolates showing virulence genes of typical EAEC and production of Shiga Toxin 2 [28].

Although virulence genes were detected and directly associated with the *E. coli* load, all four samples contained low counts of *E. coli*, ranging from 2.7 to 3.8 MPN mL ^−1^. Therefore, even though all cells had virulence genes, the microbiological load present would not be sufficient to make a healthy person ill. DuPont et al. [29] tested the virulence of 2 *E. coli* samples in experimental in vitro and in vivo models, and volunteers that ingested solutions of control strains (enterotoxigenic and enteroinvasive) in cell concentrations below 10^6^ CFU·mL^−1^ showed no symptoms. However, the ingestion dose of Enterohemorrhagic *E. coli* seems to be lower and is usually estimated to range between fewer than 10 and few hundred [30, 31]. Notably, the ingestion of *E. coli* cells with virulence genes is worrisome in infants, children with immature immune systems, and immunocompromised people [32]. Although, in terms of a public health, the microbiological load present in the aliquots was not sufficient to generate severe symptoms resulting from the clinical course caused by enteroinvasive and enterotoxigenic strains; the detection of these genes in the samples demonstrates the urgent need for hygienic-sanitary improvements in milk processing.

At this point, it is clear that monitoring the microbial load of *E. coli* can provide valuable insights into food safety, particularly for individuals with immune disorders, which prompts a comprehensive discussion about current international milk quality regulations. The European Union, the United States of America, and the Southern Common Market (MERCOSUR) are aligned regarding the maximum statutory limit of 10 CFU·mL^−1^, established exclusively for *Enterobacteria* in pasteurized milk. While these guidelines are sufficient for the healthy population, the results encourage new lines of research to understand the real risk posed by DEC strains to immunocompromised individuals or those with immature immune systems.

Within this framework, stringent regulations alone may not be ample to fully prevent foodborne outbreaks, even in highly developed countries. For instance, a large foodborne outbreak related to ultrahigh pasteurized milk served in school lunches occurred in 2021 in Toyama City [33]. Over 1800 children from 25 schools were reported. The causative pathogen was an atypical DEC, notably presenting a microbiological load ranging from 7.0 to 8.4 MPN 100 mL^−1^.

In this context, regulatory compliance is more effective when accompanied by preventive strategies to minimize the risk of postpasteurization contamination. Traditionally, certain quality management practices have been associated with reducing postpasteurization contamination in fluid milk, such as cleaning and sanitation protocols, personal hygiene and training, preventive maintenance of equipment, and adherence to good manufacturing practices. More recently, updated research is attempting to better describe patterns of postpasteurization contamination and predict this kind of issue through machine learning and other advanced statistical modeling [21] concluded that to truly take advantage of these analytical approaches, more frequent assessment of microbiological data and high-quality metadata is essential.

The results indicate that implementing microbiological monitoring of *E. coli*, even if not required by regulatory agencies, can aid dairy industries in preventing postpasteurization contamination issues and/or accelerate corrective measures if such issues occur, and the frequency of this monitoring could be further studied using predictive statistical models.

## 4. Conclusions

Although most pasteurized milk samples meet established quality standards, *E. coli* harboring virulence genes persist throughout the pasteurized milk production chain. Due to the direct association between bacterial load and these genetic determinants, the results of this study also highlight the importance of continuous microbiological monitoring in the dairy production chain, not only to allow inferences about the legal compliance of samples but also, more importantly, to emphasize the need for new lines of research to understand the sources of contamination and the real risk posed by pathogenic strains to immunocompromised individuals or those with immature immune systems.

Even with the limitations in generalizing the results to other regions or populations, it is reasonable to suggest advanced biosurveillance approaches along with virulence gene monitoring in dairy plants. In this context, the present study provides insights that can aid public health authorities in enhancing food safety policies [34].

## Figures and Tables

**Table 1:** Primers sequences of the target genes of diarrheagenic *E. coli* strains researched.

Target gene	Sequence	Product size (bp)	Reference
*stx1*	ATA AAT CGC CAT TCG TTG ACT AC AGA ACG CCC ACT GAG ATC ATC AC	180	[17]

*stx2*	GGC ACT GTC TGA AAC TGC TCC TCG CCA GTT ATC TGA CAT TCT G	225	[17]

*ipaH*	GTT CCT TGA CCG CCT TTC CGA TAC CGT C GCC GGT CAG CCA CCC TCT GAG AGT AC	620	[18]

*est*	TGA AAA AGC TAA TGT TGG CAA T ACA GGC AGG ATT ACA ACA AAG	203	[19]

*elt*	ATT TAC GGC GTT ACT ATC CTC TTT TGG TCT CGG TCA GAT ATG	280	[20]

*eae*	GAC CCG GCA CAA GCA TAA GC CCA CCT GCA GCA ACA AGA GG	384	[17]

*bfpA*	AAT GGT GCT TGC GCT TGC TGC GCC GCT TTA TCC AAC CTG GTA	324	[16]

*aggR*	GTA TAC ACA AAA GAA GGA AGC ACA GAA TCG TCA GCA TCA GC	254	[16]

**Table 2:** Counts (mean ± standard deviation) and nonconformity rate of microbiological parameters of 750 samples of pasteurized milk from 2019 to 2022.

	Counts (M ± SD)	Nonconformity rate^∗^
*Enterobacteriaceae* (CFU mL^−1^)	3.9 ± 16.70	4.8%
*Salmonella* spp. (CFU mL^−1^)	Absence	0%
*Escherichia coli* (MPN mL^−1^)	2.6 ± 12.10	No parameters

^∗^Based on the parameters set in the statutory Brazilian regulation for pasteurized milk.

**Table 3:** Influence of *E. coli* load on the likelihood of virulence genes presence in 28 samples of pasteurized milk contaminated with *E. coli* from 2019 to 2022.

Variable	Coefficient (β)	Standard error	*t* value	*p* value	Odds ratio	95% confidence interval
Intercept	−6.90	2.86	−2.41	0.02	0.001	−12.77; −1.02
*E. coli* load	−1.98	0.93	2.11	0.04	7.21	0.06; 3.89

## Data Availability

The datasets used and analyzed in this study are available from the corresponding author upon reasonable request.
